# Systematic Review of Dyspnea and Chronic Fatigue in Patients With Long COVID: Clinical Characteristics and Associated Laboratory Parameters

**DOI:** 10.1155/pm/5426125

**Published:** 2026-02-03

**Authors:** Maria Eduarda Souza Melo-Oliveira, Roberto Alves Lourenço, Eduardo Buzanovsky Louzada, Marcela Moutinho, Alessandra Ferrarese Barbosa, Virgílio Garcia Moreira, Luís Cristóvão Porto

**Affiliations:** ^1^ Graduate Program in Medical Sciences, Faculty of Medical Sciences, Rio de Janeiro State University, Rio de Janeiro, Rio de Janeiro, Brazil, uerj.br; ^2^ Research Laboratory on Human Aging, Internal Medicine Department, Faculty of Medical Sciences, Rio de Janeiro State University, Rio de Janeiro, Rio de Janeiro, Brazil, uerj.br; ^3^ Health Research Support Facility Center (CAPCS), Rio de Janeiro State University, Rio de Janeiro, Rio de Janeiro, Brazil, uerj.br; ^4^ Tissue Repair and Histocompatibility Technologic Core (TIXUS), Rio de Janeiro State University, Rio de Janeiro, Rio de Janeiro, Brazil, uerj.br

**Keywords:** clinical characteristics, dyspnea, fatigue, inflammatory markers, laboratory findings, long COVID-19, post-COVID-19 syndrome, pulmonary function tests

## Abstract

**Abstract:**

Dyspnea and chronic fatigue stand out as prevalent manifestations in the postacute phase of COVID, resulting in substantial adverse effects on patients′ quality of life and functional capacity. Although these symptoms have been widely documented, there is no clear consensus on the pathophysiological mechanisms that underlie them. The available literature reveals a dispersion of clinical and laboratory data, and the variability in the methods of assessment of fatigue and dyspnea, as well as in the laboratory variables examined, limits the standardized understanding of this complex condition.

**Objective:**

This study was aimed at identifying and synthesizing the evidence on the main clinical and laboratory characteristics related to dyspnea and fatigue in patients during long COVID from 2021 onwards.

**Methods:**

The main databases used to select the studies were PubMed and Medline, also using LitCovid and Embase.

**Results:**

A total of 42 articles that met the inclusion criteria were included, covering a total population of 30,682 patients diagnosed with COVID‐19. The findings underscore the significant impact of long COVID on patients′ quality of life, with persistent symptoms such as fatigue and dyspnea affecting a considerable proportion of individuals for durations ranging from 1 to 24 months.

**Conclusion:**

The heterogeneity in research approaches highlights the urgent need for collaborative initiatives to elucidate the determinants of long COVID symptomatology and create more consistent evaluation protocols.

## 1. Introduction

In March 2020, when the World Health Organization (WHO) announced the onset of the COVID‐19 pandemic, few anticipated its potential evolution into a chronic condition. Post‐COVID‐19 syndrome or long COVID, recognized by the WHO as a distinct clinical entity, manifests as long‐term effects that persist for 12 weeks or more after the acute phase of the disease has resolved [1, 2]. Currently, over 65 million individuals worldwide are dealing with the long‐term impacts of COVID‐19 [3]; 6 in every 100 people who have COVID‐19 develop post‐COVID‐19 condition [4]. Tragically, governments and civil societies worldwide have not adequately addressed the challenges posed by long COVID, particularly in lower‐income countries (The Lancet, 2023, Mar 11, editorial).

While most individuals infected with the severe acute respiratory syndrome coronavirus 2 (SARS‐CoV‐2) recover within weeks, a significant proportion continues to experience prolonged symptoms. Estimates indicate that 10%–30% of nonhospitalized patients, 50%–70% of those hospitalized [5], and 10%–12% of vaccinated individuals may develop post‐COVID‐19 syndrome [6]. This syndrome tends to predominantly affect individuals aged 36–50 and disproportionately impacts women, particularly those who have experienced mild COVID‐19 [7]. Beyond demographic factors such as age and sex, underlying health conditions such as systemic arterial hypertension, diabetes mellitus, and obesity are recognized as potential risk factors for developing post‐COVID‐19 syndrome [8].

The precise pathophysiological mechanisms underlying long COVID remain unclear. Nevertheless, ongoing research reveals a wide range of symptoms that persist for various durations. Studies suggest a potential association between the syndrome and the reactivation of SARS‐CoV‐2, either in a latent form or as residual viral components leading to chronic inflammation. Acosta‐Ampudia et al. [9] and Kervevan et al. [10] proposed that the syndrome could be linked to reactivation of SARS‐CoV‐2 in its latent state or through viral remnants, provoking an antiviral response that results in chronic inflammation, also suggested by Stein et al. [11]. Alternatively, immune dysregulation contributing to microvascular inflammation, thrombosis, and neuronal damage may play a role in the syndrome′s manifestations [12]. Additionally, immune dysregulation leading to microvascular inflammation and thrombosis may further contribute to neuronal damage [13].

Fatigue and dyspnea rank among the most reported symptoms in long COVID [14]. Fatigue is characterized by a reduction in physical and/or mental performance influenced by central, psychological, and peripheral factors. Dyspnea is experienced as difficulty in breathing and shortness of breath. Townsend et al. reported that even after 100 days from the onset of acute COVID‐19 symptoms, fatigue resembling chronic fatigue syndrome (CFS) may persist [15]. Furthermore, alterations in chest computed tomography have been noted between 60 and 100 days postacute COVID‐19 phase [16], suggesting potential long‐term effects on pulmonary health in certain patients. Studies propose that elevated T cell counts and increased levels of IL‐6, a cytokine correlated with COVID‐19 severity, may contribute to ongoing symptoms such as dyspnea and fatigue in individuals with long COVID [17]. The strongest predictive risk factors for persistent dyspnea included the following: female sex, elevated body mass index, pulmonary comorbidities, pre‐existing anxiety and depression, pre‐COVID‐19 physical limitations, the severity of the COVID‐19 illness, and socioeconomic differences. Potential risk factors included increased age, smoking history, and COVID‐19 variant type [18]. Previous systematic reviews indicated potential risk factors for post‐COVID‐19 fatigue were old age, female sex, severe clinical status in the acute phase of infection, a high number of comorbidities, and a prediagnosis of depression/anxiety [19] with a global CFS as a long COVID symptom prevalence of 45.2% [20], more recently in Sub‐Saharan Africa was 70% among health professionals [21].

Post‐COVID‐19 syndrome reveals a significant knowledge gap encompassing various levels of analysis, including clinical, pathophysiological, epidemiological, and therapeutic aspects. Despite advances in identifying symptoms and the most affected populations, the underlying mechanisms, the duration of long‐term effects, and effective therapeutic strategies remain largely unknown, highlighting the urgent need for further investigation. The pathophysiological mechanisms involved in long COVID are still obscure. Although there are hypotheses pointing to the possible reactivation of SARS‐CoV‐2 or immunological dysfunctions as causes of persistent symptoms, the biological basis of these conditions lacks proof. Further studies are essential to understand how these processes manifest in different population groups and what specific mechanisms are related to chronic inflammation and neuronal damage. The variability and scope of symptoms represent another challenge. Post‐COVID‐19 syndrome is characterized by a wide range of clinical manifestations, the intensity and duration of which vary considerably among individuals. The absence of standardized diagnostic criteria and a clear classification of these manifestations hinders both diagnosis and treatment. Future research is essential to more precisely map these symptoms in different populations, enabling a more complete understanding of the condition. Regarding long‐term health effects, COVID‐19 can leave persistent sequelae, especially in the respiratory and neurological systems. There is evidence of visible changes in imaging exams, such as chest computed tomography scans, and prolonged neuropsychiatric symptoms. However, knowledge about the prevalence, severity, and evolution of these effects is still limited, requiring continuous patient monitoring and long‐term studies. The profile of affected individuals also requires greater attention. Although risk factors related to sex, age, and comorbidities have already been identified, the causal relationship between these elements and the progression of post‐COVID‐19 syndrome is not yet fully understood. Furthermore, the influences of socioeconomic conditions and geographic context on the manifestation and outcome of long COVID are aspects that deserve more detailed investigation. Regarding treatment and therapeutic interventions, the options currently available are limited and often lack consistent scientific support. Conducting well‐structured clinical trials is essential to identify effective strategies that can alleviate symptoms and improve patients′ quality of life. Finally, the psychosocial aspects of long COVID represent an equally important dimension. The impact of this condition on mental health and the interactions between physical and psychological symptoms are not yet fully understood. Relationships between the persistence of symptoms and the emergence of anxiety, depression, and mental fatigue need to be further explored to promote integrated and holistic therapeutic approaches.

These gaps highlight the complexity of long COVID and reinforce the need for multidisciplinary research that investigates the phenomenon from different perspectives and in various population contexts. The continuity of studies and international collaboration are essential to broaden the understanding of this condition and guide more effective interventions that can minimize its impact and promote the full recovery of affected individuals.

The objectives of this review were to identify and synthesize evidence on the main clinical and laboratory characteristics associated with the presence of dyspnea and fatigue in patients with long COVID since 2021 and to analyze trends, evolutions, and divergences in the objectives of the studies over time.

## 2. Methods

### 2.1. Literature Search and Study Selection

**Figure 1:**
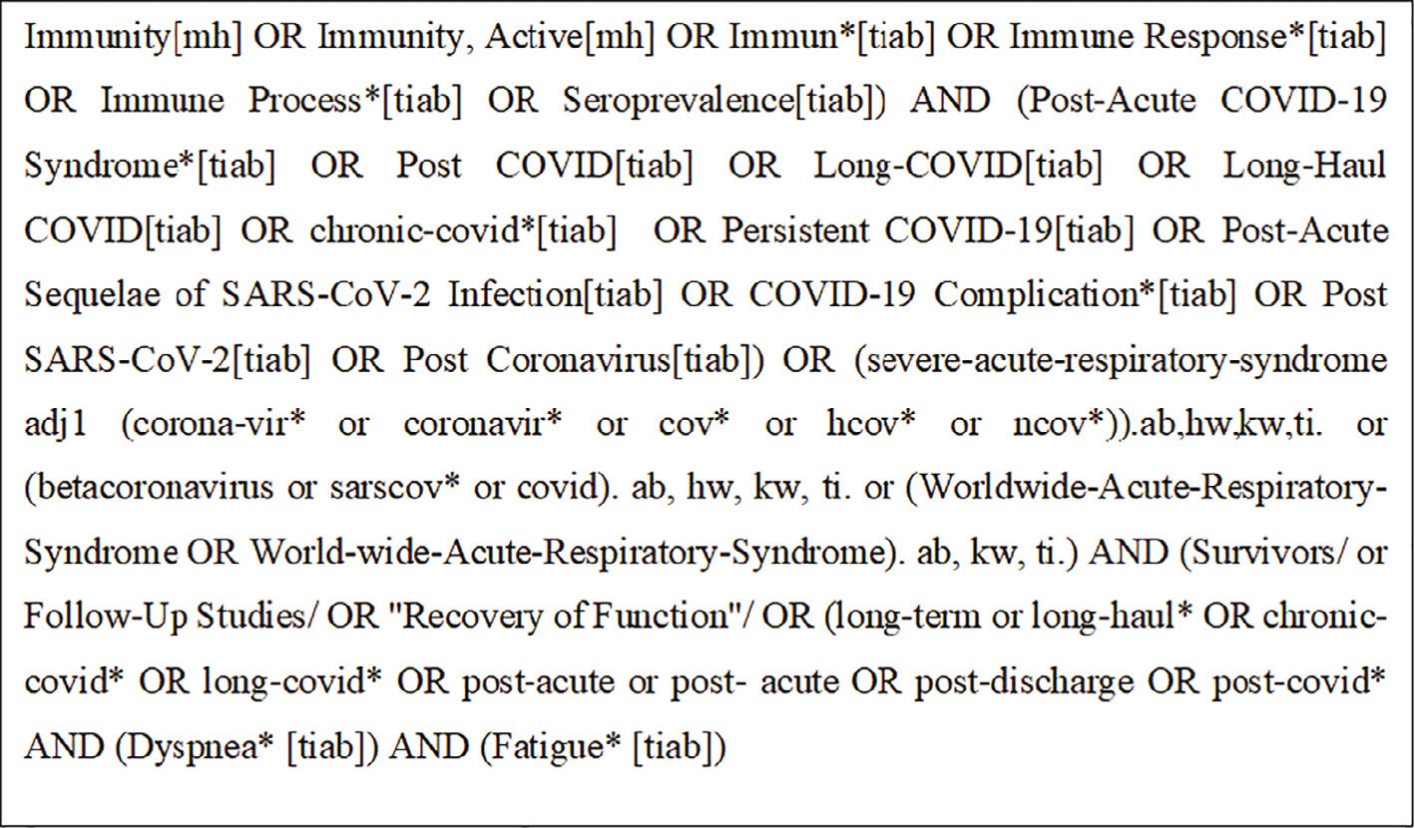
Search strategy.

This systematic review follows the guidelines of the Preferred Reporting Items for Systematic Reviews and Meta‐Analyses (PRISMA) statement, as applicable. We conducted a literature search on symptoms of dyspnea and fatigue in long COVID syndrome in February 2025. The main databases used for study selection were PubMed and Medline, through LitCovid accessed February 20, 2025 [22], and Embase (accessed on February 20, 2025). The search strategy is detailed in Figure 1.

### 2.2. Inclusion and Exclusion Criteria

We established inclusion and exclusion criteria based on the PECO model:

P (Population): Patients in the post‐COVID‐19 period diagnosed with long COVID.

E (Exposure): Presence of persistent symptoms such as dyspnea and/or fatigue.

C (Comparator): Post‐COVID‐19 patients without persistent symptoms (optional, may be omitted in descriptive reviews).

O (Outcome): Clinical characteristics (e.g., dyspnea intensity, fatigue scale, and comorbidities) and laboratory characteristics (e.g., CRP, D‐dimer, lung function, and inflammatory markers).

#### 2.2.1. Inclusion Criteria

Inclusion criteria include the following: observational studies (cohort, case–control, cross‐sectional); population: adult patients (> 18 years) with confirmed diagnosis of COVID‐19, patients in the postacute period (usually > 4 weeks after diagnosis); presence of persistent symptoms: dyspnea and/or fatigue; studies describing clinical and/or laboratory characteristics, published in English, Portuguese, or Spanish and published in the last 5 years (or since 2020).

#### 2.2.2. Exclusion Criteria

Exclusion criteria include studies with pediatric populations, reviews, editorials, letters to the editor, studies that do not distinguish persistent symptoms from the acute phase, and studies without clear clinical‐laboratory data.

After the initial search and duplicate removal, all records were imported into EndNote Version 20 (Clarivate Analytics) for further deduplication.

### 2.3. Identification of Studies

The review process employed a two‐stage screening methodology. Initially, pairs of reviewers (M.E.S.M‐O. and A.F.B.) assessed titles and abstracts using pre‐established criteria. A thorough examination of full texts followed this to evaluate their conformity with eligibility requirements. In instances of disagreement regarding the inclusion of an article, the reviewers engaged in discussions and consultations with a senior reviewer to achieve a consensus.

### 2.4. Data Synthesis

Our primary outcome of interest was to assess not only the significant increase in the quantity of articles and studies but also whether there has been an evolution in diagnostic and therapeutic approaches, alongside identifying new pathophysiological mechanisms associated with symptoms in long COVID. Given the variability in defining “Day 0” across studies, we accepted definitions that included the onset of COVID‐19 symptoms, COVID‐19 diagnosis, or hospital discharge after acute illness.

### 2.5. Quality Assessment

Reviewers independently assessed the risk of bias for each included study utilizing the Joanna Briggs Institute′s critical appraisal tool for prevalence studies. This tool consists of nine key domains that evaluate different dimensions of study quality, including sample structure, sampling method, sample size adequacy, subject and study setting descriptions, sample coverage, condition identification methods, reliability of condition measurement, appropriateness of statistical analysis, and response rate adequacy [23]. Each study was evaluated across these domains, with outcomes categorized as yes, no, or not clear. An overall score was assigned based on the number of affirmative responses.

## 3. Results

**Figure 2:**
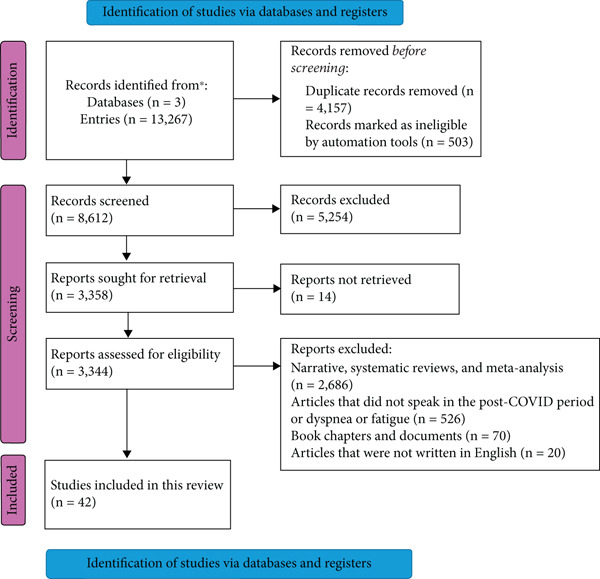
Flowchart indicating the steps to select the full papers analyzed.

A total of 13,267 abstracts were initially identified, of which 42 studies met our inclusion criteria and were included in the final analysis (Figure 2). During the review of the full texts, the main reason for exclusion of articles was the absence of data on dyspnea and fatigue symptoms during the specified follow‐up period (*n* = 5254), followed by the inclusion of patients with COVID‐19 without laboratory confirmation (*n* = 526).

Among the 42 included studies (Table 1), the total COVID‐19 population was 30,682 with 24 studies originating from Europe (sample size = 14,055), 6 from North America (sample size = 2426), 5 from South America (sample size = 1557), 3 from Asia (sample size = 901), and 1 from Oceania (sample size = 133). A study by Pazukhina et al. [57] included 11,860 participants from four continents: South America, Africa, Europe, and Asia [47]. All 42 studies have passed the quality assessment—Joanna Briggs Institute critical appraisal tool for prevalence studies—and were classified as high quality.

**Table 1:** Characteristics of included studies, year of publication, country, size of the cohort (*n*), percentage of men (male sex), age, and study type.

**Year**	**Authors**	**Country**	**n**	**Male sex (%)**	**Age** **m** **e** **a** **n** ± **S** **D** **or median (range)**	**Study type**
2021	Darcis et al. [24]	Belgium	199	63.3	60.5 ± 13.9	Monocentric prospective observational
2021	Mandal et al. [25]	United Kingdom	384	62.0	59.9 ± 16.1	Cross‐sectional study
2021	Mohiuddin Chowdhury et al. [26]	Bangladesh	313	80.2	37.7 ± 13.7	Prospective multicenter cross‐sectional
2021	Ong et al. [27]	Singapore	288	84.4	44 (33–56)	Longitudinal multicenter cohort
2021	Peluso et al. [28]	United States	121	45.5	44 (37–57)	Cohort study
2021	Scherlinger et al. [29]	France	34	40.0	40 (35–54)	Prospective study
2021	van den Borst et al. [30]	Netherlands	124	60.0	59 ± 14	Prospective observational
2022	Ballering et al. [3]	Netherlands	4231	34.3	52.4 ± 11.7	Observational cohort
2022	Bellan et al. [31]	Italy	324	60.5	61 (≥ 18)	Prospective cohort
2022	Fernández‐De‐Las‐Penãs et al. [32]	Spain	412	52.5	62 ± 15	Cohort study
2022	Ganesh et al. [33]	United States	108	25.0	46 (37–55)	Cohort study
2022	Maamar et al. [34]	Spain	121	43.8	Women 47.2 ± 13; men 45.7 ± 17	Cross‐sectional study
2022	Phetsouphanh et al. [35]	Australia	133	50.4	48.13 ± 11.89	Prospective cohort
2022	Schaeffer et al. [36]	Canada	49	26.0	50 (> 18)	Prospective study
2022	Schultheiß et al. [37]	Germany	318	39.6	51.2 (15–83)	Cohort study
2022	Staudt et al. [38]	Germany	101	58.4	60 (28–69)	Prospective study
2022	Stoffels et al. [39]	Netherlands	125	56.8	69 (≥ 18)	Observational study
2023	Beyer et al. [40]	Germany	69	23.0	46 ± 12	Cross‐sectional study
2023	Fernández‐de‐Las‐Peñas et al. [41]	Spain	1266	53.5%	61 ± 16	Multicenter cohort study
2023	Kalil‐Filho et al. [42]	Brazil	480	67.5	59 ± 14	Cohort study
2023	Kervevan et al. [10]	France	80	76.3	48.8 (33.2–60.7)	Observational cohort
2023	Mariano et al. [43]	Brazil	232	30.6	50.0 ± 12.8	Cross‐sectional study
2023	Muzyka et al. [44]	Ukraine	332	32.2	45 (28–70)	Prospective cohort
2023	Núñez et al. [45]	Mexico	192	65.3%	53 (44–64)	Prospective cohort study
2023	Paradowska‐Nowakowska et al. [46]	Poland	471	42.9%	63.83 ± 9.93	Cross‐sectional and retrospective study
2023	Román‐Montes et al. [47]	Mexico	246	54.0%	50 (41–63)	Cross‐sectional study
2023	Xuereb et al. [48]	United Kingdom	249	40.0%	46.1 ± 13.8	Case–control study
2024	Areekal et al. [49]	India	300	52.7%	44.95 ± 15.5	Cross‐sectional study
2024	Azambuja et al. [50]	Brazil	710	32.0%	41 (31–53)	Prospective cohort study
2024	Cornelissen et al. [51]	Netherlands	95	50.6%	53.9 ± 6.1	Multicenter, prospective, observational study
2024	Floridia et al. [52]	Italy	1297	48.5%	59.6 ± 13.9	Multicenter cohort study
2024	Gutiérrez‐Canales et al. [53]	Mexico	68	61.8%	50.93 ± 14.98	Observational, prospective, and longitudinal analytical study
2024	Ida et al. [54]	Brazil	58	38.0%	52.8 ± 10.5	Prospective study
2024	Kirchberger et al. [55]	Germany	304	41.8%	52 (40–59)	Prospective observational study
2024	Parás‐Bravo et al. [56]	Spain	76	59.6%	69 ± 10.5	Cross‐sectional study
2024	Pazukhina et al. [57]	Four continents	11,860	47.9%	52 (41–62)	Prospective and observational study
2024	Santos et al. [58]	Brazil	77	47.0%	55 ± 11.8	Cross‐sectional study
2024	Smith et al. [59]	Canada	1642	35.4	49.1 ± 13.6	Cross‐sectional study
2024	Steinke et al. [60]	Germany	1810	18.0%	48 ± 12.2	Bidirectional cohort study
2025	Hatakeyama et al. [61]	Japan	220	71.40%	57 (50–61)	Multicenter cohort study
2025	Peter et al. [62]	Germany	1558	34.2%	48.1 ± 12.3	Prospective, multicenter, observational, case–control study
2025	Ülker Ekşi et al. [63]	Türkiye	75	37.3%	21.3 ± 1.86	Cross‐sectional study

Table 2 shows that the articles analyzed share similar objectives, focusing on investigating the proportion of patients who present persistent symptoms or who develop them after COVID‐19 infection. In addition, they seek to analyze the prevalence of long COVID, its impact on health, and the risks associated with comorbidities, as well as to evaluate clinical and physiological variables in patients facing persistent fatigue and dyspnea. The duration of symptoms ranged from 1 to 24 months, revealing a worrying picture that significantly affects the quality of life of many individuals. Studies indicate that approximately 20% of patients still report long COVID 6 months after infection, a condition that is particularly prevalent among women and people with comorbidities, who face greater challenges in their recovery and report lower levels of functioning and quality of life. The data demonstrate that patients diagnosed with this syndrome face limitations in physical capacity, both in maximal and submaximal assessments, severely impacting their daily activities. Furthermore, the evolution of symptoms, such as fatigue and dyspnea, is shown to fluctuate and can persist for up to 2 years after infection, highlighting the complexity and heterogeneity of the syndrome, which varies according to sex, the phase of the pandemic, and the severity of the acute disease. The most common symptoms include generalized fatigue, arthralgia, dyspnea, anxiety, and sleep disorders, affecting cognitive, emotional, and motor functions.

**Table 2:** Authors, objective of the study, post‐COVID‐19 time, measurements, and results of the study.

**Author**	**Objectives**	**Long-COVID follow-up**	**Measurements**	**Results**
Darcis et al. [24]	To determine the persistent consequences of COVID‐19	3–6	Blood tests	High and stable prevalence of persistent symptoms up to 6 months postdischarge
Mandal et al. [25]	To follow up on all SARS‐CoV‐2‐positive COVID‐19 admissions 4–6 weeks after discharge	1–2v	Persistent symptoms, biomarkers, chest imaging	Identifying persistent symptoms and radiological abnormalities suggests the need for additional rehabilitation and investigation
Mohiuddin Chowdhury et al. [26]	Investigate acute and postrecovery manifestations of COVID‐19 illness, post‐COVID‐19 persisting symptoms/complaints, and changes in lab parameters	5	Initial hematological and radiology findings, post‐COVID‐19 symptoms/complaints, lab changes	21.4% of symptomatic patients experienced persistent symptoms > 20 weeks, along with lab changes
Ong et al. [27]	Investigate clinical symptoms and immune cytokine profiles post‐COVID‐19	3–6	Clinical symptoms, radiologic data, plasma samples for immune mediators	Approximately 10% of recovered patients had persistent symptoms 6 months after infection, with immune cytokine signatures reflecting inflammation
Peluso et al. [28]	Compare fold changes in markers between those with and without PASC	3	Blood samples, persistent immune activation assessment	Persistent immune activation is associated with ongoing symptoms post‐COVID‐19
Scherlinger et al. [29]	Investigate underlying mechanisms, incl. autoimmunity and psychological distress	1	Questionnaires for symptom evaluation, blood tests	Revealed burden experienced by patients with long‐term COVID‐19 symptoms and possible underlying mechanisms
van den Borst et al. [30]	Assess health domains in patients 3 months postrecovery from acute COVID‐19	1–2	Lung function, CT/x‐ray, 6‐min walking test, body composition, health questionnaires	Severe health issues in ex‐COVID‐19 patients require longer follow‐up studies
Ballering et al. [3]	Analyze the nature, prevalence, and severity of long‐term COVID‐19 symptoms	3–6	Digital questionnaires, symptom assessment	Around one in eight patients had persistent symptoms after COVID‐19, considering symptom severity and other factors
Bellan et al. [31]	Identify risk factors for COVID‐19 sequelae in hospitalized subjects followed up 1 year after discharge	12	Multidisciplinary approach, clinical and lung function assessment, mini‐international neuropsychiatric interview, proinflammatory cytokine analysis	Persistent proinflammatory state associated with long COVID, with IL‐12 playing a central role
Fernández‐De‐Las‐Penãs et al. [32]	Investigate serological biomarkers′ association with post‐COVID fatigue and dyspnea	6–12	Laboratory	Some serological biomarkers are associated with post‐COVID fatigue or dyspnea without a complete explanation of long‐COVID problems
Ganesh et al. [33]	Describe clinical findings from the first patients seen in a post‐COVID care clinic	5	Blood tests, inflammation markers, autoimmune screening, 6‐min walk test, pulmonary function	Elevated IL‐6 levels are linked to postacute sequelae of SARS‐CoV‐2 infection, with gender differences in symptom presentation
Maamar et al. [34]	Evaluate inflammatory markers in subjects with PCS after mild COVID‐19	3	Blood tests	Association between PCS and elevated inflammatory markers observed
Phetsouphanh et al. [35]	Define immunological parameters associated with long COVID	8	Blood tests	Sustained inflammatory response post‐COVID, with possible multiple etiologies
Schaeffer et al. [36]	Hypothesize that CPET and symptom ratings identify differences in COVID survivors with persistent fatigue	3	Self‐reported questionnaires, blood tests, echocardiography, pulmonary function tests	Lower fitness linked to post‐COVID fatigue; psychological burden amplifies symptom perception
Schultheiß et al. [37]	Provide evidence for a long‐lasting cytokine signature potentially underlying the clinical symptom of PASC	10	Basic and PASC‐specific questionnaires, blood tests	Chronic elevation of IL‐1*β*, IL‐6, and TNF in patients with PASC was observed
Staudt et al. [38]	Evaluate symptoms and physiological deficiencies in hospitalized patients after 10 months	10	Functional assessments, depression assessments, clinical data collection	High prevalence of symptoms like shortness of breath, fatigue, and cognitive impairment 10 months postdischarge
Stoffels et al. [39]	Explore quadriceps muscle weakness in post‐COVID‐19 cohorts	12	Lung function, BMI, body composition, nutritional status, physical and lung capacity assessment	High prevalence of muscle weakness post‐COVID hospitalization, with identified risk factors
Beyer et al. [40]	To investigate exercise capacity and markers of subjective well‐being and their independent relationship with post‐COVID‐19 syndrome	≥ 3	Exercise capacity, quality of life, and fatigue	Patients with post‐COVID‐19 syndrome present reduced maximal and submaximal physical performance, as well as limitations in quality of life, particularly pronounced in the physical components
Fernández‐de‐Las‐Peñas et al. [41]	To apply Sankey plots and exponential bar charts to visualize the evolution and trajectory of post‐COVID fatigue and dyspnea symptoms in a cohort of previously hospitalized COVID‐19 survivors	18	Evolution and trajectory of post‐COVID fatigue and dyspnea symptoms	The use of Sankey charts shows a fluctuating evolution of post‐COVID fatigue and dyspnea during the first 2 years after infection
Kalil‐Filho et al. [42]	Evaluate cardiopulmonary symptoms and related images in discharged COVID‐19 patients	3	Demographics, clinical history, lab tests, imaging	Common and multifactorial cardiopulmonary symptoms appeared 90 days postdischarge
Kervevan et al. [10]	Characterize humoral and CD4+ T cell responses in long COVID patients	3	Cell and humoral immune response assays	Divergent findings suggest multiple etiologies in long COVID cases
Mariano et al. [43]	Evaluate clinical, spirometric characteristics according to the severity of prior COVID‐19 infection	12	Age, sex, hospitalization, symptoms	Restrictive ventilation disorder and pneumonia are associated with severe initial conditions
Muzyka et al. [44]	Identify physiology‐centered risks, symptoms, and lab findings in post‐COVID‐19 patients	1	Lifestyle questionnaires, autoimmune response tests, inflammatory markers	Prevalence of long COVID at 23%, with age, lifestyle, and inflammatory markers influencing the risk and manifestations observed
Núñez et al. [45]	To provide detailed clinical phenotyping, including onset, duration, and co‐occurrence of persistent symptoms, to better understand post‐COVID conditions in an understudied population. In addition, we analyzed a set of clinical covariates to determine their association with post‐COVID conditions	3	Presence of post‐COVID‐19 conditions in people in Latin America	In our cohort, symptoms of post‐COVID conditions were frequent (particularly respiratory and neurocognitive) and persistent. Importantly, prior vaccination against SARS‐CoV‐2 resulted in a shorter duration of post‐COVID conditions
Paradowska‐Nowakowska et al. [46]	To assess the frequency and correlations of prolonged COVID‐19 symptoms with gender, disease severity, time since disease onset, and exercise capacity in the Polish convalescent population hospitalized as part of a comprehensive cardiopulmonary rehabilitation (CR) program after COVID‐19	6	Long COVID symptoms with gender, disease severity, time since disease onset, and exercise capacity	Increased fatigue and dyspnea correlate with impaired spirometric parameters and significantly affect the exercise capacity of convalescents
Román‐Montes et al. [47]	To describe the prevalence, characteristics, and impact on quality of life of post‐COVID‐19 syndrome in patients with a history of hospitalization for COVID‐19	4	Quality of life and symptoms of patients post‐COVID	Post‐COVID syndrome occurred in 76% of hospitalized patients with a prolonged duration and impaired QoL
Xuereb et al. [48]	To investigate symptoms and health‐related quality of life in the medium‐term follow‐up (6 months post‐COVID)	6	Medium and long‐term cardiovascular sequelae	Post‐COVID‐19 patients present persistent symptoms in medium‐term follow‐up
Areekal et al. [49]	To determine the proportion of patients who have persistent symptoms or develop new symptoms 6 months after COVID‐19 infection and determine the associated factors, among those discharged from a tertiary care center in South India	6	Persistent symptoms	The study concludes that one‐fifth of patients still suffer from post‐COVID‐19 syndrome even 6 months after COVID‐19 infection
Azambuja et al. [50]	To determine the prevalence of long COVID, its health impact, and associated risk factors in one such community in Rio de Janeiro, Brazil	3	Persistent symptoms and quality of life	Women and individuals with comorbidities were more likely to report poorer recovery, functioning, dyspnea, and quality of life
Cornelissen et al. [51]	Determine the occurrence of fatigue and other symptoms and assess how many patients meet the criteria for myalgic encephalomyelitis/chronic fatigue syndrome	9–12	Prevalence of fatigue in post‐COVID	This study shows persistent fatigue and diverse symptomatology in post‐COVID‐19 patients, up to 12–18 months after SARS‐CoV‐2 infection
Floridia et al. [52]	To assess, in patients accessing care for long COVID, the profile of reported symptoms, the possible clustering of symptoms and cases, the functional status compared to preinfections, and the impact on work activity	1	The grouping of symptoms of post‐COVID patients	The findings provide further evidence that long COVID is a heterogeneous disease with manifestations that differ by sex, pandemic phase, and severity of acute illness and support the possibility that multiple pathways lead to different clinical manifestations
Gutiérrez‐Canales et al. [53]	To describe the persistence of COVID‐19 symptoms and QoL among patients 3 and 12 months after hospital discharge	3–12	Post‐COVID syndrome and its impact on quality of life	As the proportion of persistent symptoms at 12 months is high, we suggest that patients should continue long‐term follow‐up to reclassify, diagnose, and treat new‐onset symptoms/diseases
Ida et al. [54]	To describe the persistent symptoms of post‐COVID‐19 syndrome (especially neurological symptoms) and their cognitive, emotional, motor, quality of life, and indirect cost repercussions 12 months after infection	12	The negative impact on health, quality of life, and productivity of post‐COVID syndrome	The most frequent persistent symptoms in people with post‐COVID‐19 syndrome who sought rehabilitation at the Fortaleza unit of the SARAH network refer to generalized fatigue, arthralgia, dyspnea, anxiety, depression, and sleep disturbances, impacting cognitive, emotional, motor function and the quality of life of patients
Kirchberger et al. [55]	To compare the quality of life and mental health of individuals with and without post‐ COVID syndrome in a German sample of people not hospitalized after SARS‐CoV‐2 infection, to characterize the long‐term course up to 2 years, and identify predictors for post‐COVID‐19 disabilities	24	Quality of life and mental health of patients post‐COVID	Post‐COVID syndrome in nonhospitalized individuals following SARS‐CoV‐2 infection is often associated with long‐term impairments in mental health and health‐related quality of life outcomes
Parás‐Bravo et al. [56]	To compare serological biomarkers between patients with ILD who develop and those who do not develop post‐COVID‐19 symptoms	12	Post‐COVID‐19 symptomatology in patients with interstitial lung disease	The presence of post‐COVID‐19 fatigue or dyspnea was also associated with higher creatine phosphokinase levels in patients with idiopathic interstitial lung disease
Pazukhina et al. [57]	Characterize long COVID, explore the risk factors for developing long COVID, and the impact on daily activities and quality of life in different regions	2–12	COVID‐19 sequelae reported in low‐ and middle‐income countries	Our data show that long COVID affects populations globally, manifesting similar symptomatology and impact on functioning in both high‐income countries and LMICs. Prevalence was higher in HICs than in low‐ and middle‐income countries
Santos et al. [58]	To assess clinical and physiological variables in patients with post‐COVID‐19 condition and persistent fatigue	12	Clinical variables of fatigue in post‐COVID	Those with persistent fatigue had a greater sensation of dyspnea, higher levels of anxiety, reduced peripheral and inspiratory muscle strength, and greater impairment of quality of life. The severity of fatigue was influenced by worsening quality of life, increased levels of anxiety, and decreased peripheral muscle strength
Smith et al. [59]	Identify factors that are independently associated with this symptom	3	Clinically phenotype those with dyspnea associated with post‐COVID conditions	We speculate that there are at least two phenotypes associated with dyspnea: A phenotype with pronounced fatigue (normal PFT) and a phenotype with pronounced lung abnormalities (abnormal PFT)
Steinke et al. [60]	To determine the prevalence and trajectory of persistent symptoms after COVID‐19 and to investigate factors influencing them among employees in health and welfare services in Germany	8–13	Prevalence and symptoms in post‐COVID syndrome	More than a year after a COVID‐19 illness, two‐thirds of healthcare staff surveyed reported persistent symptoms
Hatakeyama et al. [61]	To investigate the prevalence of functional impairments and the effects of long COVID on long‐term functional impairments in patients with severe COVID‐19	12	Functional impairments and the effects of long COVID	Many patients with severe COVID‐19 admitted to the ICU still suffered from postintensive care syndrome even after a year, which manifested in combination with direct symptoms of the original disease, such as long COVID
Peter et al. [62]	To describe clinical characteristics and diagnostic findings among patients with PCS persisting for > 1 year and assess risk factors for persistence vs. improvement of PCS	> 12	The long‐term prognosis of these postacute sequelae of COVID‐19/post‐COVID‐19 syndrome	We observed that most working‐age patients with PCS did not recover in the second year of their illness. Reported symptom patterns remained similar, nonspecific, and dominated by fatigue, exercise intolerance, and cognitive complaints
Ülker Ekşi et al. [63]	To investigate respiratory and functional sequelae in young adults with asymptomatic or mild COVID‐19 compared with healthy peers, 3–6 months and 6–12 months after COVID‐19 infection	3–12	Respiratory and functional sequelae in young adults post‐COVID	Young adults who experience asymptomatic or mild SARS‐CoV‐2 infection experience a decline in FVC% predicted, PEF% prev, lower limb muscle performance, and physical function within 3–6 months

The data also confirm that long COVID was not limited to hospitalized patients; those who did not require hospitalization also experienced a significant deterioration in their mental health and quality of life, with a worrying prevalence of persistent symptoms up to 18 months after infection. Prior vaccination against SARS‐CoV‐2 appears to be associated with a shorter duration of long COVID symptoms, suggesting that this factor could be considered in management strategies for the condition.

The analysis of patients with persistent fatigue revealed a correlation with increased dyspnea and reduced muscle strength, factors that contribute to limitations in physical capacity. The severity of fatigue is strongly linked to a decrease in quality of life and increased anxiety, highlighting the complexity of the syndrome through phenotypes related to dyspnea, which can manifest with varied clinical characteristics, some factors also depicted in a previous systematic review [18].

In comparison, articles published since 2023 have focused on the influence of inflammatory biomarkers, the investigation of clinical symptoms and immunological cytokine profiles in long COVID‐19, and the search for underlying mechanisms, including autoimmunity and psychological impact. The study period ranged from 1 month to 1 year. The results of these investigations also highlight the severity of the situation, highlighting the need for monitoring and appropriate clinical interventions. The prevalence of persistent symptoms remains high and stable, with approximately 21.4% of symptomatic patients presenting persistent conditions for more than 20 weeks after infection, often accompanied by laboratory alterations.

Approximately 10% of patients considered recovered continue to report symptoms 6 months after infection, with immune cytokine profiles indicating a residual inflammatory state. Persistent immune activation is associated with the persistence of long COVID symptoms, revealing a significant impact on patient health. Symptoms such as dyspnea, fatigue, and cognitive impairment remain common up to 10 months after hospital discharge, and approximately one in eight patients may experience persistent symptoms depending on their severity.

The persistent proinflammatory state, linked to long COVID, may be evaluated by the determination of interleukin‐12 (IL‐12). Serological biomarkers have been shown to be associated with long COVID fatigue or dyspnea. Elevated levels of IL‐6 are correlated with acute sequelae of SARS‐CoV‐2 infection, and gender differences influence how symptoms manifest. Studies show a consistent association between long COVID and elevated inflammatory markers, suggesting a sustained inflammatory response after COVID‐19 infection with other comorbidities.

Furthermore, decreased physical capacity is correlated with long‐COVID fatigue, and psychological factors may amplify the perception of symptoms. The findings revealed chronic elevations of cytokines such as IL‐1*β*, IL‐6, and TNF in patients with long COVID, in addition to a high prevalence of muscle weakness after hospitalization for COVID‐19, with several risk factors identified. Multifactorial cardiopulmonary symptoms were observed 90 days after hospital discharge, and the divergent findings suggest that several etiologies may be involved in long COVID cases. With a prevalence of long COVID estimated at 23%, factors such as age, lifestyle, and inflammatory markers play significant roles in determining the risk and manifestations observed. These findings highlight the importance of a comprehensive and continuous approach in managing the consequences of COVID‐19.

Since 2023, the predominance of questionnaires as an assessment tool in the selected studies highlights a change in the approach to investigating long COVID, especially regarding symptoms such as fatigue, dyspnea, and patients′ quality of life. Thus, most researchers appear to have emphasized participants′ self‐assessment of their conditions, allowing for a more subjective and individualized understanding of the patient′s experience. Only two studies chose to integrate imaging tests, which suggests a tendency to prioritize direct clinical assessment over diagnosing structural changes. On the other hand, five studies applied lung function tests, further evidencing the concern with the objective assessment of respiratory capacity, which is fundamental to better understanding the long‐term implications of COVID‐19. Laboratory tests were another important tool, with particular emphasis on the analysis of biomarkers such as C‐reactive protein (CRP), lymphocytes, and hemoglobin, which can provide valuable information about the inflammatory state and immune function of patients. In contrast to the first search, where a greater use of imaging tests was observed, the use of questionnaires was less frequent, with only seven studies employing this assessment methodology. At that time, the priority was more focused on discovering objective evidence through imaging and laboratory tests, totaling 16 articles that investigated various markers, with a special emphasis on inflammatory cytokines. This difference in methodological approaches indicates an evolution in research on long‐COVID, where there is now a greater appreciation of the patient′s perspective and an attempt to understand not only the physical manifestations of the disease but also its impact on quality of life. This transition reflects an important advance in the understanding and management of long‐COVID, suggesting that the field is adapting to the needs and experiences of affected individuals. Comparing the methodologies between the two searches highlights not only the dynamics of scientific research but also the complexity of COVID‐19‐related conditions, which require multiple approaches for a more comprehensive understanding of their lasting effects (Table 3).

**Table 3:** Questionnaires, exams, and laboratory markers of studies.

**Author**	**Chest CT**	**Laboratory**	**Spirometry and other tests**	**Questionnaire**
Darcis et al. [24]	CXR	Platelets, lymphocytes, D‐dimer, ferritin, creatinine, ALT and AST, glucose, CRP	ND	ND
Mandal et al. [25]	Chest CT	Hemoglobin, erythrocyte, white blood cell, red blood cells, platelets, CRP, SGPT, ferritin, prothrombin, D‐dimer, creatinine	ND	ND
Mohiuddin Chowdhury et al. [26]	CXR	ND	ND	ND
Ong et al. [27]	ND	MCP‐1, IL‐6, IL‐10, TNF‐*α*, IFN‐*γ*‐induced protein 10, IFN‐*γ*, immunoglobulin G	ND	ND
Peluso et al. [28]	ND	IL‐1*β*, IL‐2, IL‐4, IL‐5, IL‐6, IL‐8, IL‐10, IL‐12p70, IL‐13, IL‐17A, IL18, IFN‐*γ*, IP‐10, G‐CSF, GM‐CSF, MCP‐1, MIP‐1*α*, MIP‐1*β*, TNF‐*α*	ND	ND
Scherlinger et al. [29]	ND	Hemoglobin, D‐dimer, creatinine, GGT, and CRP	Plethysmography	6MWT, PHQ‐9‐D, body, DL, SGRQ
van den Borst et al. [30]	Chest CT	ND	Plethysmography	mMRC, 6MWT, SF‐36, HADS, PTSS, DSM‐5, DL, body
Ballering et al. [3]	ND	ND	ND	SCL‐90, SOM
Bellan et al. [31]	ND	IFN‐*γ*, IL‐1*β*, IL‐2, IL‐6, IL‐12, IL‐17, TNF‐*α*	Spirometry	Mini mental
Fernández‐De‐Las‐Penãs et al. [32]	ND	Hemoglobin, lymphocyte, neutrophil, platelet, glucose, CRP, CK, LDH, D‐dimer, ALT, and AST	ND	ND
Ganesh et al. [33]	Chest CT	Complete blood, comprehensive metabolic panel, ESR, CRP, ferritin, D‐dimer, IL‐6	Spirometry	6MWT
Maamar et al. [34]	·ND	PCR, neutrophil, lymphocyte, NLR, lactate, ferritin, fibrinogen, D‐dimer	ND	ND
Phetsouphanh et al. [35]	·ND	Interferon *β*, IFN‐*λ*1, IFN‐*γ*, CXCL9, CXCL10, IL‐8, IL‐6, IL‐33	ND	ND
Schaeffer et al. [36]	·ND	PCR, D‐dimer, vitamin D	Transthoracic echocardiography, spirometry, CPET	mMRC, HADS, IER‐S, PPS, D‐12, Borg scale
Schultheiß et al. [37]	ND	TNF‐*α*, LTA, TNF‐*β*, IL‐1*β*, IL‐4, IL‐6, IL‐8, IL‐13, IFN‐*α*2	ND	ND
Staudt et al. [38]	ND	ND	ND	ND
Stoffels et al. [39]	Chest CT	PCR, D‐dimer, ferritin	Spirometry	PG‐SGA, SF, mMRC, SF‐36, 6‐MWT, DL
Beyer et al. [40]	ND	ND	ND	FAS, 6MWT, HADS, SF‐36
Fernández‐de‐Las‐Peñas et al. [41]	ND	ND	ND	Patients were systematically questioned via telephone interview about self‐reported symptoms of fatigue and dyspnea developed after hospitalization
Kalil‐Filho et al. [42]	Chest CT	Troponin I, D‐dimers, PCR, creatinine	ND	EQ‐5D, GAD‐2, PHQ‐2
Kervevan et al. [10]	ND	IL‐2, IFN‐*γ*, TNF‐*α*, CD3+ CD4+ CD8−	ND	ND
Mariano et al. [43]	Chest CT	ND	Spirometry	ND
Muzyka et al. [44]	ND	D‐dimers, PCR, fibrinogen, blood cells	ND	ND
Núñez et al. [45]	ND	ND	ND	An 84‐item questionnaire was used to collect data on clinical symptoms during telephone calls
Paradowska‐Nowakowska et al. [46]	ND	ND	ND	6MWD
Román‐Montes et al. [47]	ND	ND	ND	EQ‐5D, PCS, VAS
Xuereb et al. [48]	ND	ND	ND	SF‐36
Areekal et al. [49]	ND	ND	ND	Schedule a semistructured interview over telephone interview
Azambuja et al. [50]	ND	ND	ND	EQ‐5D‐5L, WG‐SS MRC
Cornelissen et al. [51]	ND	ND	ND	FSS, DSQ‐2
Floridia et al. [52]	ND	ND	ND	Short version of the post‐COVID‐19 case report form (CRF) from the WHO global clinical platform for COVID‐19
Gutiérrez‐Canales et al. [53]	ND	ND	ND	SF‐36, PHQ‐9, GAD‐7
Ida et al. [54]	ND	ND	ND	HADS, MoCA, SF‐36, PCFS, 6MWT, hand grip, TUG, FSS
Kirchberger et al. [55]	ND	ND	ND	VR‐12, PHQ‐D, IES‐R, FAZ
Parás‐Bravo et al. [56]	CT	Lactate dehydrogenase, creatine phosphokinase, leukocytes, neutrophils, lymphocytes, alanine transaminase, aspartate transaminase, glucose, hemoglobin, platelets, D‐dimer, albumin, sodium, potassium	ND	ND
Pazukhina et al. [57]	ND	ND	ND	A standardized case report form developed by the global COVID‐19 follow‐up working group of the International Severe Acute Respiratory and Emerging Infection Consortium assessed the frequency of fever, persistent symptoms, shortness of breath (MRC dyspnea scale), fatigue, and impact on daily activities
Santos et al. [58]	ND	ND	ND	Hand grip, SGRQ, HADS, mMRC, 6MWD
Smith et al. [59]	ND	ND	ND	PCFS, 6MWD, 5D‐5L, PHQ‐9, GAD‐7
Steinke et al. [60]	ND	ND	ND	The baseline questionnaire contained items on sociodemographic data, height, weight, smoking status, exercise habits, subjective health status, and occupational information. Pre‐existing medical conditions and retrospective data on acute COVID‐19 illness were recorded
Hatakeyama et al. [61]	ND	ND	ND	MRC, HADS, PDS, TIL scale, EQ‐5D‐5L
Peter et al. [62]	ND	ND	ND	mMRC hand grip, PSQI, ISI, ESS, CFQ‐11, SF‐12, PHQ‐9, GAD‐7
Ülker Ekşi et al. [63]	ND	ND	ND	6MWD, BORG, FSS, hand grip, TLS, IPAQ‐SF, ISWT

Abbreviations: 6MWT, Six‐Minute Walk Test; ALP, alkaline phosphatase; ALT, alanine aminotransferase; AST, aspartate aminotransferase; BORG, Borg Rating of Perceived Exertion scale; CFQ‐11, Chalder Fatigue Scale; COD (DLCO), diffusing capacity of the lung for carbon monoxide; CRP, C‐reactive Protein; CT, computed tomography; DSM‐5, Diagnostic and Statistical Manual of Mental Disorders, Fifth Edition; DSQ‐2, DePaul Symptom Questionnaire‐2; EQ‐5D, EuroQol 5 Dimensions; EQ‐5D‐5L, EuroQol 5 Dimensions, 5 Levels; ESS, Epworth Sleepiness Scale; FAS, Fatigue Assessment Scale; FEV1, forced expiratory volume in 1 second; FSS, Fatigue Severity Scale; FVC, forced vital capacity; GAD‐2, Two‐Item Generalized Anxiety Disorder Scale; GAD‐7, Seven‐Item Generalized Anxiety Disorder Scale; GFR, glomerular filtration rate; GGT, gamma‐glutamyl transferase; Hb, hemoglobin; HbA1, glycated hemoglobin; HADS, Hospital Anxiety and Depression Scale; HDL, high‐density lipoprotein; hs‐CRP, high‐sensitivity C‐reactive protein; IFN‐γ, interferon gamma; IL‐6, interleukin 6; IPAQ‐SF, International Physical Activity Questionnaire–Short Form; ISI, insomnia severity index; ISWT, Incremental Shuttle Walk Test; IES‐R, Impact of Event Scale–Revised; LDH, lactate dehydrogenase; LDL, low‐density lipoprotein; MEP, maximum expiratory pressure; MIC, minimum inhibitory concentration; MIP, maximum inspiratory pressure; mMRC, Modified Medical Research Council Dyspnea Scale; MoCA, Montreal Cognitive Assessment; MPV, mean platelet volume; ND, not available; PCFS, Post‐COVID‐19 Functional Status Scale; PDS, Posttraumatic Diagnostic Scale; PEF, peak expiratory flow; PHQ‐9, Patient Health Questionnaire‐9; PHQ‐D, Patient Health Questionnaire; PSQI, Pittsburgh sleep quality index; RDW, red cell distribution width; SCL‐90, Symptom Checklist‐90; SF‐12, Short Form‐12 Health Questionnaire; SF‐36, Short Form Health Survey; SGRQ, St. George′s Respiratory Questionnaire; TIL Scale, Three‐Item Loneliness Scale; TLC, total lung capacity; TLS, One‐Minute Sit‐to‐Stand Test; TLS5x, Five Times Sit‐to‐Stand Test; TNF‐α, tumor necrosis factor alpha; TSH, thyroid‐stimulating hormone; TUG, Timed Up and Go; VAS, Visual Analogue Scale; VR‐12, Veterans RAND 12‐Item Health Survey.

The main comorbidities identified over a year of research reveal remarkable consistency in their nomenclature and frequencies (Table 4). Hypertension stood out as one of the most prevalent conditions, recorded in 10 articles in the initial analysis and rising to 14 in the most recent studies. This trend not only suggests a worrying public health pattern but also emphasizes the need for continued monitoring of this condition among patients who have faced or are recovering from COVID‐19. Diabetes also consolidated itself as a significant comorbidity, being mentioned in 13 articles in the first assessment and increasing to 15 in the second, which reinforces the critical intersection between this condition and the persistent effects of the infection. In addition, cardiac problems were reported in 10 articles, highlighting the concern about cardiovascular health as a direct or indirect complication of COVID‐19, indicating the need for effective prevention and control strategies.

**Table 4:** Main comorbidities.

**Author**	**Arterial hypertension (%)**	**Diabetes (%)**	**Obesity (%)**	**Asthma/COPD/CLD (%)**	**Chronic heart disease/cardiovascular (%)**
Darcis et al. [24]	44.2%	36.2%	36.20%	ND	ND
Mandal et al. [25]	40.4%	26.3%	ND	16.9%	9.4%
Mohiuddin Chowdhury et al. [26]	6.7%	ND	ND	4.2%	0.6%
Ong et al. [27]	18.1%	9.4%	ND	ND	4.5%
Peluso et al. [28]	ND	11.6%	ND	19.0%	ND
Scherlinger et al. [29]	8.8%	29.4%	ND	ND	ND
van den Borst et al. [30]	27.4%	13.7%	ND	38.7%	24.2%
Ballering et al. [3]	ND	ND	ND	ND	ND
Bellan et al. [31]	ND	ND	ND	ND	ND
Fernández‐De‐Las‐Penãs et al. [32]	19.4%	7.3%	3.60%	2.9%	10.2%
Ganesh et al. [33]	ND	13.9%	38.90%	17.6%	10.2%
Maamar et al. [34]	ND	ND	28.60%	ND	ND
Phetsouphanh et al. [35]	28.0%	15.2%	ND	38.4%	20.0%
Schaeffer et al. [36]	42.1%	22.5%	ND	8.5%	29.6%
Schultheiß et al. [37]	ND	1.3%	3.80%	3.8%	2.5%
Staudt et al. [38]	40.6%	9.9%	ND	9.9%	11.9%
Stoffels et al. [39]	44.0%	21.1%	20.70%	ND	ND
Beyer et al. [40]	ND	ND	ND	ND	ND
Fernández‐de‐Las‐Peñas et al. [41]	ND	ND	ND	ND	ND
Kalil‐Filho et al. [42]	ND	ND	ND	ND	ND
Kervevan et al. [10]	ND	ND	ND	ND	ND
Mariano et al. [43]	ND	ND	ND	ND	ND
Muzyka et al. [44]	ND	ND	ND	ND	ND
Núñez et al. [45]	36.1%	36.1%	ND	6.9%	10.4%
Paradowska‐Nowakowska et al. [46]	50.7%	17.6%	ND	8.1%	12.7% CAD, 3.8% AMI
Román‐Montes et al. [47]	33%	23%	43%	3% asthma, 2% COPD	4%
Xuereb et al. [48]	22%	10%	39%	16%	0.6%
Areekal et al. [49]	ND	ND	ND	ND	ND
Azambuja et al. [50]	20%	5.9%	32%	3.3%	ND
Cornelissen et al. [51]	ND	14.3%	ND	6.6% COPD, 16.5% asthma	6.6%
Floridia et al. [52]	42.6%	11.3%	16.5%	6.6%	6.6%
Gutiérrez‐Canales et al. [53]	45.6%	27.9%	35.3%	4.4%	ND
Ida et al. [54]	43%	28%	28%	ND	ND
Kirchberger et al. [55]	21.1%	4.6%	ND	ND	2.3% AM, 15.3% CAD
Parás‐Bravo et al. [56]	ND	ND	ND	ND	ND
Pazukhina et al. [57]	22%	10%	10%	6.7% asthma, 3.9 COPD	6.0%
Santos et al. [58]	ND	ND	ND	ND	ND
Smith et al. [59]	ND	ND	ND	12.3% asthma, 1.8% COPD	ND
Steinke et al. [60]	ND	ND	ND	13%	26%
Hatakeyama et al. [61]	28.2%	26.4%	ND	4.6%	1.8%
Peter et al. [62]	ND	ND	ND	16.7%	22.9%
Ülker Ekşi et al. [63]	ND	ND	ND	ND	ND

*Note:* The studies of Maamar et al. [34], Phetsouphanh et al. [35], Schultheiß et al. [37], and Muzyka et al. [44] did not provide information on comorbidities. Autoimmune diseases: 9—16.40% in Peluso et al.; oncologic: 20—16.10% in van den Borst et al. and 2—2.50% in Kerkevan et al.; immunocompromised: 18—14.50% in van den Borst et al.; allergy: 34—42.50% in Kerkevan et al.; chronic diseases: 397—10.40% in Ballering et al.

Abbreviation: NR, not reported in the publication.

Asthma and chronic obstructive pulmonary disease (COPD) were also frequently mentioned in the studies, highlighting the importance of close monitoring for these groups, given the vulnerability associated with respiratory conditions that can be exacerbated by viral infection. Obesity, which was initially addressed in 6 articles, maintained its relevance by being cited in 9 subsequent studies, showing the importance of weight management in patient recovery and post‐COVID interventions. Notably, only 2 articles in the first analysis and 3 in the second did not discuss the comorbidities of the participants, which highlights the indisputable relevance of these risk factors in the context of the research.

The continued presence of these comorbidities not only underscores the complexity of the clinical picture of patients dealing with the lasting effects of COVID‐19 but also emphasizes the need for integrated and multidisciplinary approaches to treatment and rehabilitation. Such an approach should include a focus on preventive care and the management of underlying conditions, contributing to a more effective recovery and an improvement in the quality of life of affected patients. Identifying and monitoring these comorbidities is crucial for developing intervention strategies that can mitigate their consequences and improve health outcomes for individuals facing the postinfectious challenge.

## 4. Discussion

The COVID‐19 pandemic has brought to light a few public health challenges, including the emerging condition known as long COVID, characterized by persistent symptoms that impact the quality of life of many survivors. This systematic review analyzed data from 42 observational studies obtained in an initial search and 21 in a search 1 year later, focusing on participants with confirmed COVID‐19, diagnosed through positive SARS‐CoV‐2 tests or direct medical evaluations. The results of the comparison between the two searches provide critical insights into persistent dyspnea and fatigue in post‐COVID settings, highlighting the importance of quantitative and qualitative analyses in understanding this new condition. The prevalence of persistent symptoms, such as fatigue and dyspnea, is especially concerning in the context of post‐COVID care. Analysis of the studies reveals that most patients experience a significant deterioration in their quality of life due to the prolonged effects of the infection. Early in the pandemic, Mandal et al. [25] emphasized the need for systematic monitoring after hospital discharge, warning that many patients are discharged without adequate assessment of possible sequelae. The lack of rehabilitation strategies combined with inadequate identification of persistent symptoms hinders the recovery of these individuals, resulting in not only physical but also psychological impacts. On the other hand, in a study by Abdel‐Gawad et al. [64], the authors highlight the need to reassess the current literature to better understand the clinical spectrum associated with SARS‐CoV‐2. Regarding the representativeness of the data, the comparative analysis between the first and second searches reveals a significant increase in the inclusion of participants: In the first search, there were 6600 in Europe, 990 in the Americas, 1591 in Asia, and 133 in Oceania. In the second search, these numbers increased to 8786 in Europe, 2724 in the Americas, and 3244 in Asia, while Oceania remained unchanged. This growth, especially in the Americas and Asia, suggests an increase in awareness of long COVID and an evolution in global recognition of the problem. Greater representativeness of publications in the Americas and Asia indicates a movement toward a more comprehensive understanding of the condition, reflecting the diversity of experiences around the world.

To investigate the trajectory and emerging understanding of long COVID, dyspnea, and fatigue, we conducted a systematic review. The selected articles before 2023 reflected the preliminary knowledge regarding these prevalent symptoms. More recent publications highlighted not only a significant increase in the volume of articles and research but also notable advancements in diagnostic and therapeutic strategies, as well as the identification of new pathophysiological mechanisms related to these symptoms.

To better understand the symptoms of dyspnea and fatigue in long‐COVID‐19, we conducted a systematic literature review, allowing us to analyze possible trends, evolutions, and divergences in the objectives of the studies over time of nuances in the approaches to symptoms of dyspnea and fatigue, widely recognized as critical manifestations of long‐COVID. Publications before 2023 addressed clinical characteristics and secondary laboratory findings related to these symptoms, seeking to integrate a comprehensive view of the impact of COVID‐19 on the respiratory health and functional capacity of affected individuals. However, more recent publications approached methods to evaluate health status of individuals and the frequency of symptoms in response to a growing body of evidence and discussions about long COVID. This interval allowed for divergence of criteria and expansion of investigations into clinical aspects and laboratory results, highlighting the importance of a continuous analysis of the nuances surrounding the syndrome. The comparison between these two periods not only highlighted the evolution of scientific understanding of long COVID but also highlighted areas in which new research priorities have emerged, such as the identification of relevant biomarkers and the efficacy of different intervention methods. Thus, this review contributes not only to the understanding of long COVID‐related dyspnea and fatigue symptoms but also to the construction of scientific knowledge that can guide future clinical interventions and management strategies for scrutiny of dyspnea or fatigue among these patients. The methodological approach of these publications also presents significant differences. In publications before 2023, the focus was on objective assessments carried out between 1 and 12 months postinfection, using blood tests and lung function tests. In contrast, the recent ones extended this interval to up to 24 months and incorporated questionnaires that assessed not only patients′ quality of life but also their emotional aspects. This transition reflects an important shift toward a patient‐centered approach that seeks to understand the subjective experience of health and the challenges faced after infection.

Studies such as those by Areekal et al. [49] and Azambuja et al. [50] corroborate that approximately 20% of patients continue to report persistent symptoms up to 6 months after infection. These findings emphasize that fatigue and dyspnea are not only common symptoms but that they have a profound impact on the daily lives of affected individuals. The research by Beyer et al. highlights the relationship between the decline in exercise capacity and the reduction in quality of life and physical performance, reinforcing the need for effective rehabilitation strategies.

In addition, the persistence of symptoms not only affects individual well‐being but has significant implications for patients′ daily functioning and work capacity. Fernandez de la Penas et al. [41] and Hatakeyama et al. [61] observed significant variations in functional recovery, revealing an alarming picture regarding persistent fatigue and other sequelae related to COVID‐19. Identifying these symptoms and developing appropriate interventions is essential, not only for the patient′s recovery but also to prevent future complications.

The analysis also reveals that demographic factors and comorbidities play a crucial role in the severity and persistence of symptoms. Geographic location influences the prevalence and diversity of symptoms; developing countries may face greater challenges due to lack of health infrastructure. Gutiérrez‐Canales et al.′s [53] study highlighted the interrelationship between comorbidities and mental health problems, such as anxiety and depression, which further impact the quality of life of patients with chronic conditions.

The research by Floridia et al. [52] and Hatakeyama et al. [61] reaffirms the need for long‐term monitoring of those who develop long COVID, recognizing that many individuals may continue to experience significant symptoms more than a year after initial infection, which indicate that approximately 12.7% of survivors still experience persistent symptoms, highlighting the urgency of implementing targeted clinical interventions and ongoing support.

In addition, it is important to highlight the economic impact associated with persistent symptoms. Studies show that the consequences of absenteeism are significant, implying high indirect costs, which become a critical concern for public health systems. The analysis by Ida et al. [54] showed that the cost of absenteeism on productivity is directly related to the health of COVID‐19 survivors and the need for policies that ensure appropriate interventions and ongoing support.

Since 2023, greater emphasis on posthospitalization care reflects the different definitions of long COVID that have been adopted globally. The increasing number of studies conducted in COVID outpatient clinics, as opposed to specialty outpatient clinics, suggests a necessary focus on ongoing care and early interventions, as many patients are discharged from hospital without proper assessment of their condition. This is crucial, as effective management of COVID‐19 sequelae depends on recognizing and appropriately treating these manifestations. Although there has been a significant evolution in the understanding and management of long COVID and the presence of dyspnea or fatigue, the need for an interdisciplinary approach is clear, as is the urgency of implementing health policies that ensure continuous support for these patients.

This study presents important strengths and some limitations that deserve consideration. Among the positive aspects, the temporal updating of the review, carried out at two distinct times, stands out, allowing for observation of the evolution of scientific knowledge about long COVID and the expansion of the geographical representativeness of the included studies. The approach adopted is comprehensive, encompassing not only clinical aspects but also psychological, functional, and socioeconomic impacts, demonstrating a multidimensional understanding of the syndrome. Furthermore, the study highlights the methodological transition from purely laboratory analyses to patient‐centered approaches, incorporating quality of life assessments and emotional aspects, reflecting a relevant conceptual advancement. The identification of knowledge gaps, such as the need for specific biomarkers and effective rehabilitation strategies, also represents a significant contribution to the field. However, some limitations must be acknowledged. The heterogeneity of the included observational studies limits the strength of the conclusions presented. There is also an inequality in regional representativeness, with a scarcity of data from Oceania and an absence of African studies, which compromises the generalizability of the results. Finally, the potential publication bias and the predominantly descriptive nature of the findings indicate the need for future reviews with greater methodological standardization and consolidated statistical analysis. Nonetheless, the efficacy of treatment in hospitalized patients with mild to moderate COVID‐19 [65] and their impact in long COVID was not evaluated in this study. Despite these limitations, the work contributes significantly to the understanding of long COVID, especially regarding the persistence of symptoms such as dyspnea and fatigue, and reinforces the importance of public policies and interdisciplinary approaches focused on the care and rehabilitation of affected patients and emphasizes the responsibility of health systems in addressing this emerging public health problem.

## 5. Conclusion

In conclusion, long COVID represents an emerging and highly relevant challenge for global public health, reflecting a worrying perspective on the quality of life of survivors. This systematic review highlighted the significant persistence of symptoms, such as fatigue and dyspnea, in a considerable proportion of patients, even months after the initial infection. The results demonstrate that demographic factors, the presence of comorbidities, and mental health conditions exert a substantial influence on the severity and clinical presentation of symptoms, highlighting the existing inequalities between different population groups. The prolonged maintenance of these clinical manifestations not only compromises the physical and psychological well‐being of individuals but also generates significant socioeconomic impacts, such as increased absenteeism from work and reduced productivity. Thus, it becomes imperative to develop and implement integrated management approaches that articulate efforts between health professionals, researchers, and public policymakers, with a view to developing therapeutic and rehabilitative strategies directed at the multiple domains affected by post‐COVID‐19 syndrome. This analysis reinforces the need for systematized protocols for follow‐up and rehabilitation after hospital discharge, ensuring that all survivors receive continuous assessment and adequate multidisciplinary support. An interdisciplinary approach is therefore essential to address the complexities inherent in long COVID, promoting evidence‐based interventions that can reduce its long‐term impacts and optimize the quality of life of those affected. The implications of this study broaden the current body of knowledge on the prolonged consequences of SARS‐CoV‐2 infection, corroborating the multifactorial and persistent nature of this condition. From a clinical point of view, these findings reinforce the need to incorporate systematic screening and multidimensional assessment of symptoms into postinfection follow‐up protocols, with an emphasis on the integration between primary care, physical rehabilitation, and mental health support. Furthermore, the evidence presented provides support for the formulation of clinical guidelines and public policies aimed not only at managing persistent symptoms but also at mitigating their functional, psychosocial, and economic effects.

## Conflicts of Interest

The authors declare no conflicts of interest.

## Funding

The study is supported by Fundação Carlos Chagas Filho de Amparo à Pesquisa do Estado do Rio de Janeiro, 10.13039/501100004586 (E‐26/210.184/2020, E‐26/211.133/2021, and E‐26/200.530/2023); Conselho Nacional de Desenvolvimento Científico e Tecnológico, 10.13039/501100003593 (310885/2022‐1); and Coordenação de Aperfeiçoamento de Pessoal de Nível Superior, 10.13039/501100002322.

## Data Availability

The data that support the findings of this study are available from the corresponding author upon reasonable request.
